# Giant cell tumors of the distal ulna: long-term recurrence rate and functional outcomes of *en bloc* resection versus curettage in a multicenter study

**DOI:** 10.1186/s13018-023-04150-4

**Published:** 2023-09-30

**Authors:** Lenian Zhou, Juan Tang, Longxiang Shen, Zhichang Zhang, Ting Yuan

**Affiliations:** 1 Department of Orthopaedics, Shanghai Sixth People’s Hospital Affiliated to Shanghai Jiao Tong University School of Medicine, 600 Yishan Road, Shanghai, 200233 China; 2 Department of Pathology, Shanghai Sixth People’s Hospital Affiliated to Shanghai Jiao Tong University School of Medicine, 600 Yishan Road, Shanghai, 200233 China

**Keywords:** Giant cell tumor of bone, Distal ulna tumors, Wrist, Darrach procedure, Sauvé–Kapandji procedure, Extensor carpi ulnaris tenodesis

## Abstract

**Objective:**

The wrist is the second most commonly involved location for GCTB, while distal ulna is a relatively rare location and limited evidence exists on which surgical approaches and reconstruction techniques are optimal. We carried out a multicenter retrospective study to evaluate the recurrence rate of distal ulna GCTB and the long-term functional outcomes of different surgery options.

**Methods:**

All 28 patients received surgical treatment for distal ulna GCTB in one of three tertiary bone tumor centers between May 2007 and January 2021 with a minimum two-year follow-up. Surgical options included intralesional curettage or *en bloc* resection (one of 3 types). Functional outcomes were assessed by the MSTS score, the QuickDASH instrument, MWS, and MHQ according to the latest treatment.

**Results:**

Overall recurrence rate was 14.2%. The curettage group (*N* = 7) had a significantly higher recurrence rate compared to *en bloc* resection (*N* = 21) (42.9% vs 4.8%) (mean follow-up: 88.8 mo). Seven patients received the Darrach procedure, 5 received the original Sauvé–Kapandji procedure, and 9 received the modified Sauvé–Kapandji procedure with extensor carpi ulnaris (ECU) tenodesis. Of the 4 patients having a recurrence, 1 received the Darrach EBR, 2 received the modified Sauvé–Kapandji procedure, and 1 received resection for soft tissue recurrence. Only MWS and esthetics in the MHQ scores were different (curettage, Darrach, Sauvé–Kapandji, and Sauvé–Kapandji with ECU tenodesis [MWS: 96.5 ± 1.3 vs 91.5 ± 4.7 vs 90.8 ± 2.8 vs 91.5 ± 3.6; esthetics in MHQ: 98.5 ± 3.1 vs 89.9 ± 4.7 vs 93.8 ± 4.4 vs 92.6 ± 3.8], respectively).

**Conclusions:**

*En bloc* resection for distal ulna GCTB had a significantly lower recurrence rate compared with curettage and achieved favorable functional outcome scores. Given the higher recurrence rate after curettage, patients should be well informed of the potential benefits and risks of selecting the distal radioulnar joint-preserving procedure. Moreover, reconstructions after tumor resection of the ulna head do not appear to be necessary.

**Supplementary Information:**

The online version contains supplementary material available at 10.1186/s13018-023-04150-4.

## Background

Giant cell tumor of bone (GCTB) is an aggressive neoplasm typically occurring near joints in young individuals [1]. As an intermediate tumor rarely associated with pulmonary metastasis, it accounts for approximately 5% of all primary bone tumors [2]. Intralesional curettage combined with locally applied adjuvants is the treatment of choice for preservation of the native joint [3]. However, with the curettage approach, local recurrence rate is still high (15–50%). When local recurrence occurs, wide *en bloc* resection can be carried out when necessary [4, 5].

After the knee, the wrist is the second most common location for GCTB to occur [6, 7]. The complex anatomy of the wrist and the aggressive behavior of this tumor makes surgical management more challenging, which can confer a poor prognosis [6, 7]. In contrast to the distal radius, the distal ulna is a relatively rare location for GCTB to occur. When GCTB does occur distally, that part of the ulna is considered to be expendable during resection. If bone is removed, it allows the remaining portion of the ulna to serve as a pseudarthrosis. Thus, supination and pronation of the forearm can still be performed after *en bloc* resection without further reconstruction [8].

The Darrach procedure has historically been reserved to treat degenerative conditions of the distal ulna [9]. Harness and Mankin [10] reported good functional outcome with some minor instability in three patients who received Darrach resections of GCTB of the distal ulna. Several techniques have also been developed to reconstruct the wrist after resection in order to improve functional outcome. These include soft tissue stabilization (extensor carpi ulnaris [ECU] tenodesis [11–13]) and bone graft procedures (Sauvé–Kapandji procedure [14]). However, it is controversial whether any type of reconstruction is needed. Most previous studies employing reconstructions were limited to cases with heterogeneous tumors and case series with small sample sizes [8, 15]. These considerations prompted us to conduct a multicenter retrospective cohort study comprising the largest series of patients to date with GCTB of the distal ulna.

In the present retrospective study, we asked the following research questions: (1) What is the local recurrence rate after intralesional curettage versus *en bloc* resections for GCTB of the distal ulna? (2) Do functional outcomes differ between patients who received *en bloc* resection with reconstruction versus those who received *en bloc* resection without reconstruction? (3) What is the range of complications after treating GCTB with different surgical techniques?

## Methods

### Patients and setting

We reviewed a total of 28 patients who had a GCTB of the distal ulna and who were surgically treated between May 2007 and January 2021 (12 men and 16 women) in three tertiary bone tumor centers. This retrospective study was approved by the Institutional Review Board of all three hospitals; all patients gave their informed consent.

### Surgery and postoperative rehabilitation

**Fig. 1:**
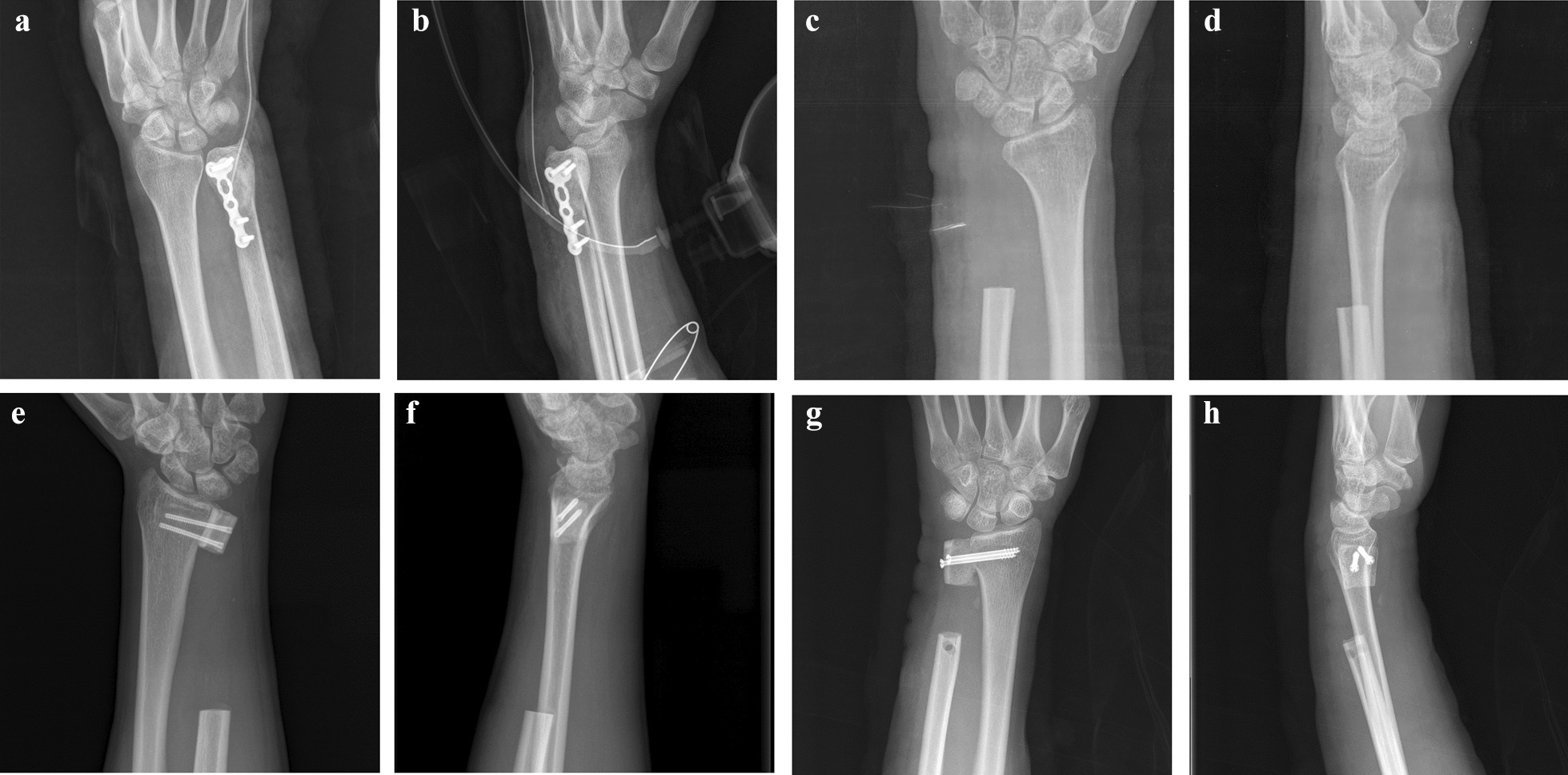
**A**, **B** Postoperative X-ray images of extended intralesional curettage. **C**, **D** Postoperative X-ray images of the Darrach procedure. **E**, **F** Postoperative X-ray images of the original Sauvé–Kapandji procedure. **G**, **H** Postoperative X-ray images of the modified Sauvé–Kapandji procedure with ECU tenodesis

The primary surgery consisted of one of four possible different procedures. Seven patients received extended intralesional curettage (Fig. 1A, B) [16], 7 patients received the Darrach procedure (Fig. 1C, D) [17], 5 patients received the original Sauvé–Kapandji procedure (Fig. 1E, F) [14], and 9 patients received the modified Sauvé–Kapandji procedure with ECU tenodesis (Fig. 1G, H) [18]. The choice of surgical method was decided after informing the patient of available treatment options and their related risks and benefits. Patients with less advanced GCTB lesions (e.g., well-maintained bony and articular architecture, and no soft tissue extension or pathologic fracture) tended to undergo extended intralesional curettage. *En bloc* resection was typically recommended for patients at potential high risk for local recurrence.

Extended intralesional curettage was carried out as previously described [19], and the resulting space was filled with a bone graft after curettage (4 patients received synthetic bone and 3 received allograft). Five patients received supplemental internal fixation, with the aim of preventing a subsequent fracture. For patients who underwent the Darrach procedure or the Sauvé–Kapandji procedure, a wide *en bloc* resection was made with a > 1 cm clear margin as planned preoperatively from the imaging and biopsy tract. The resection plan encompassed the triangular fibrocartilage complex (TFCC), the ulnar border of the pronator quadratus muscle, and the distal radioulnar joint (DRUJ) capsule. Intraoperative frozen sections were conducted to verify sufficient margins. For patients who underwent the original or modified Sauvé–Kapandji procedure, reconstruction was done with arthrodesis of the distal radioulnar in order to create a distal ulnar pseudarthrosis. In 7 of these patients, the iliac crest served as the graft for reconstructing the ulnar head, and in another 7 patients, the excised segment of the ulna was used as the graft. In this study, none of the patients received the anti-RANKL monoclonal antibody denosumab at any time as part of the treatment.

Postoperative rehabilitation was tailored to the individual patient’s needs and restrictions; it was administered under the guidance of the surgeon and physiotherapists.

### Follow-up and functional assessments

Typically, patients received follow-ups in the clinic, and radiographs were made. Radiographs were recommended at 1, 3, 6, and 12 months postoperatively for the first year, and then annually thereafter. Owing to the long-term nature of this study, the final follow-up would be carried out via telephone calls or through the WeChat messaging application (Tencent Corp., Shenzhen, Guangdong, China).

For this study, we used widely used functional scores for the final follow-up. Upper extremity function was assessed with the following tests: the Musculoskeletal Tumor Society Scoring System (MSTS) [20], QuickDASH instrument [21], Mayo Wrist Score (MWS) [22], and Michigan Hand Outcomes Questionnaire (MHQ) [23]. The latter two tests were used to evaluate specific wrist outcomes. The QuickDASH is a shortened 11-item version of the DASH (Disabilities of the Arm, Shoulder, and Hand). The MHQ is a 37-item questionnaire that is divided into six distinct subscales similar to the QuickDASH: overall hand function, activities of daily living, pain, work performance, esthetics, and patient satisfaction [23]. The MSTS and MWS were administered by the attending physician, and the QuickDASH and MHQ were completed by the patients.

### Oncologic assessment and statistical analysis

We assessed oncologic outcome by surgical type according to the first surgical treatment performed in our institution (intralesional curettage vs *en bloc* resection). Functional outcomes and complications organized by the different procedures were recorded for the most recent treatment received (intralesional curettage vs Darrach procedure vs original Sauvé–Kapandji procedure vs modified Sauvé–Kapandji procedure with ECU tenodesis).

Statistical analyses were performed using SPSS software (version 26; IBM, Armonk, NY, USA). All *p*-values were two-sided, and a value of *p* < 0.05 was taken as statistically significant. The Student’s t-test was used to analyze continuous variables, and the Pearson Chi-square test or Fisher’s exact test were used to compare differences between categorical variables. The nonparametric Kruskal–Wallis test for analysis of variance (ANOVA) with Dunn's multiple comparisons test was used to determine statistically significant differences in functional outcome scores of the patients and the different surgical procedures.

## Results

**Fig. 2:**
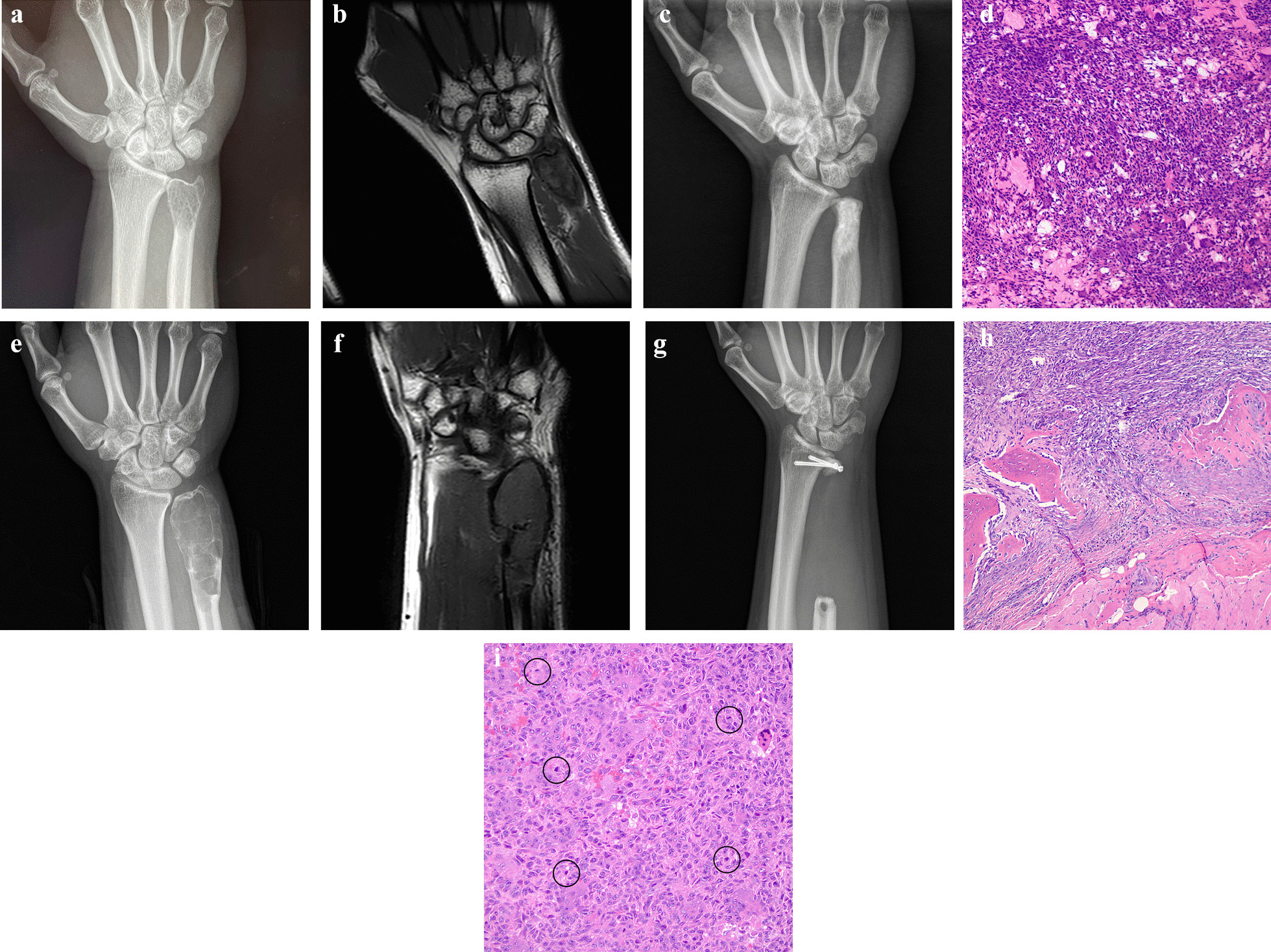
GCTB of the right distal ulna in a 36-year-old male patient. **A**, **B** X-ray and MRI images, respectively, of a GCTB in the head of the ulna. **C** X-ray of the same wrist one year after curettage. There was no sign of local recurrence. **D** Postoperative pathology showed classic histologic features of GCTB (H&E staining). **E**, **F** The patient was not followed up thereafter until injuring his wrist 131 mo after initial curettage surgery. X-ray and MRI images at 131 mo showed a recurrent GCTB with expansive growth. **G** The patient finally underwent the modified Sauvé–Kapandji procedure with ECU tenodesis to treat the recurrent GCTB. **H**, **I** Postoperative pathology revealed the tumor cells had invaded the bone cortex and mitosis was obviously visible (black circle)

Clinical and demographic characteristics of patients who were treated for GCTB are presented in Table 1 and Additional file 1. The average patient age was 32.7 years (range 17–57 years); the mean time to follow-up was 88.8 months (range 24–188 months). In 12 patients, the GCTB involved the dominant wrist. fThe overall recurrence rate was 14.3% (4/28). The incidence of local recurrence was significantly greater with intralesional curettage (3/7 or 42.9%) compared to *en bloc* resection (1/21 or 4.8%, *p* = 0.038) (Table 1). The mean time for recurrence was 54.0 mo (range 17–131 mo). Of the 4 GCTB patients experiencing recurrence, 1 received the Darrach procedure, 2 received the modified Sauvé–Kapandji procedure with ECU tenodesis, and 1 received resection for soft tissue recurrence (Fig. 2). Patients who underwent *en bloc* resection all received wide resections.

**Table 1:** Association between GCTB patients’ (*n* = 28) clinical and demographic characteristics and initial surgery treatment

Variable	Initial surgery type		*p*-value
	Intralesional curettage	*En bloc* resection	
Sex, count (%)			
Male	5 (71.4)	7 (33.3)	0.10
Female	2 (28.6)	14 (66.7)	
Campanacci classification, count (%)			
Grade II	5 (71.4)	5 (23.8)	0.063
Grade III	2 (28.6)	16 (76.2)	
Pathologic fracture, count (%)			
Yes	1 (14.3)	6 (28.6)	0.64
No	6 (85.7)	15 (71.4)	
Primary or recurrent, count (%)			
Primary	7 (100.0)	18 (85.7)	0.55
Recurrent	0 (0.0)	3 (14.3)	
Side, count (%)			
Left	3 (42.9)	13 (61.9)	0.42
Right	4 (57.1)	8 (38.1)	
Local recurrence, count (%)			
Yes	3 (42.9)	1 (4.8)	**0.038**
No	4 (57.1)	20 (95.2)	
Age, year (mean ± SD)	36.0 ± 8.0	31.5 ± 12.3	0.38
Size of tumor, cm (mean ± SD)	2.6 ± 0.5	3.3 ± 1.4	0.22
Follow-up, no. months (mean ± SD)	102.0 ± 59.3	84.4 ± 41.1	0.39

Bold values indicate *p* values are taken as statistically significant

At the final follow-up, mean QuickDASH scores of the intralesional curettage group were statistically indistinguishable from those of the groups receiving the Darrach procedure or *en bloc* resection (either original Sauvé–Kapandji procedure or the modified Sauvé–Kapandji procedure with ECU tenodesis) (Table 2). The same group pattern emerged from an analysis of the MSTS scores and the MHQ scores; no group was significantly different from the others (Table 2). Only the overall MWS scores of the intralesional curettage group were significantly higher than those of the groups receiving the Darrach procedure or *en bloc* resection (96.5 vs 91.5 vs 90.8 vs 91.5, respectively; *p* = 0.027) (Table 2).

**Table 2:** Functional outcome scores of GCTB patients according to final surgery type

Functional outcomes (mean ± SD)	Final surgery				*p*-value
	Intralesional curettage (*n* = 4)	Darrach procedure (*n* = 8)	Original Sauvé–Kapandji procedure (*n* = 5)	Modified Sauvé–Kapandji procedure with ECU tenodesis (*n* = 11)	
MSTS	28.3 ± 0.5	26.3 ± 1.4	26.0 ± 1.4	26.3 ± 1.2	0.052
QuickDASH	6.8 ± 2.7	13.4 ± 6.3	14.1 ± 4.9	12.0 ± 4.5	0.11
MWS	96.5 ± 1.3	91.5 ± 4.7	90.8 ± 2.8	91.5 ± 3.6	**0.027**
MHQ	97.4 ± 1.9	92.9 ± 4.2	92.9 ± 2.5	93.6 ± 2.8	0.12
Subscales of MHQ					
Overall hand function	96.3 ± 4.8	92.5 ± 6.0	91.0 ± 4.2	91.4 ± 5.0	0.41
ADL	98.0 ± 1.8	94.8 ± 2.4	93.8 ± 1.9	95.2 ± 2.9	0.10
Work performance	97.5 ± 2.9	94.4 ± 4.2	93.0 ± 2.7	94.5 ± 3.5	0.31
Pain (reversed)	96.3 ± 2.5	94.4 ± 5.0	92.0 ± 4.5	94.5 ± 2.7	0.42
Esthetics	98.5 ± 3.1	89.9 ± 4.7	93.8 ± 4.4	92.6 ± 3.8	**0.030**
Satisfaction	97.9 ± 2.4	91.7 ± 5.5	94.0 ± 2.1	93.2 ± 4.7	0.13

Bold values indicate *p* values are taken as statistically significant

Nonparametric Kruskal–Wallis test for analysis of variance (ANOVA) with Dunn's multiple comparisons test

ADL, activities of daily living; ECU, extensor carpi ulnaris; MHQ, Michigan Hand Outcomes Questionnaire; MSTS, Musculoskeletal Tumor Society Scoring System (MSTS); MWS, Mayo Wrist Score; QuickDASH, shortened 11-item version of the Disabilities of the Arm, Shoulder, and Hand instrument

An analysis of the subscale scores of the MHQ showed that overall hand function, activities of daily living (ADL), pain, work performance, and patient satisfaction were statistically indistinguishable among the groups (Table 2). However, the MHQ esthetic subscale score of the intralesional curettage group was significantly greater than that of the Darrach procedure group (98.5 vs 89.9, respectively; Kruskal–Wallis test, adjusted *p* = 0.019).

One patient treated with intralesional curettage experienced a postoperative complication (i.e., bone graft rejection and wound infection), whereas 5 patients receiving *en bloc* resection experienced postoperative complications, including symptoms associated with the irritation of the dorsal sensory branch of the ulnar nerve (*n* = 2), dorsal displacement of the distal ulnar stump (*n* = 1), loose screw (*n* = 1), and resorption of autogenous iliac bone graft (*n* = 1). Two patients underwent a second operation to remove screws. None of the patients experienced lung metastases, and none of them died.

## Discussion

GCTB, characterized as an intermediate and locally aggressive tumor, exhibits local recurrence rates ranging from 15 to 50% after intralesional curettage [4, 5]. Typically, these recurrences manifest within two years post-surgery [24]. Recurrence or voluminous lesions could have the increased risk to develop pulmonary metastasis, for which wide resection and reconstruction are recommended [25, 26]. GCTB of the wrist may have a poor prognosis compared with GCTB at other sites [6, 16]. However, the prognostic results of different treatment procedures are unknown due to the rarity of distal ulna GCTB and lack of relevant studies [27]. It is essential to balance curative oncologic control and maintenance of limb function. Given the “expendability” of the distal ulna, the optimal surgical approach at this site could be different from the optimal treatment for GCTB of long bones [27]. The goals of this study were to identify: (1) local recurrence rates, (2) functional outcomes, and (3) complications.

In our series of patients, we observed an overall local recurrence rate of 14.3% for GCTB of the distal ulna. One explanation for this relatively low recurrence rate is that 75% (21/28) of our patients received *en bloc* resection. The proportion is high compared to that reported in studies of GCTB of other anatomic sites. It was consistent with the treatment of choice for GCT in the proximal fibula [28]. It is our experience that patients and surgeons tend to choose *en bloc* resection for GCTB treatment because of a lower risk of local recurrence in management of aggressive tumors, especially when the site is considered to be “dispensable” bone [29]. It is important to note that the average elapsed time to recurrence in our study was 54 months, which is much longer than previously reported recurrence times [30]. Also it might reflect less severe early symptoms or signs of recurrence for patients with tumors in non-weight-bearing bones [31].

Our results indicated that there were significantly higher local recurrence rates in patients treated with intralesional curettage than those treated with *en bloc* resection (42.9% vs 4.8%, respectively; *p* = 0.038). Similarly, Jamshidi et al. [27] reported a higher recurrence rate after curettage (33%) of proximal fibula tumors compared to resection (0%). Moreover, for the three patients who experienced recurrence after curettage, their DRUJ could not be preserved because the tumors were aggressive. In addition, because the tumors of these three patients were classified as Campanacci Grade III tumors with prominent bony destruction, repeated curettage was likely not indicated to a certain extent. Recurrence of GCTB not only could lead to extra economic burden and pain for patients because of re-operation, but also might lead to higher risk of potential malignant transformation and metastasis [32, 33]. Thus, intralesional curettage should be cautiously considered for treatment of GCTB of the distal ulna and adopted only if the patients are at low risk for local recurrence and are well informed of the potential benefits and risks of this DRUJ-preserving procedure.

In our series, we did not detect an overall significant difference in functional outcome scores for patients the different surgical treatments. With the exception of certain worse MSTS scores in the intralesional curettage group, the loss of DRUJ with or without soft tissue stabilization and the bone graft procedures did not seem to adversely affect function. The Darrach procedure, an *en bloc* resection of the distal ulna, is an accepted treatment to address painful DRUJ with rheumatoid arthritis or to treat tumors of the distal ulna [8, 17]. The potential disadvantages of the Darrach procedure include loss of grip strength, instability of the proximal ulnar stump, and ulnar translocation of the carpus [8, 34]. Attempts at improvements has led to the conception of the Sauvé–Kapandji procedure. This was realized by adding an arthrodesis of the DRUJ, combined with a distal ulnar pseudarthrosis on the Darrach procedure [14]. Nevertheless, painful radioulnar impingement caused by instability of the proximal ulnar stump might still occur, for which subsequent surgical stabilization of soft tissue has been developed to stabilize the proximal ulnar stump [11, 13].

Aasheim and Finsen [35] reported average QuickDASH scores ranging from 7 to 10 for the general population of 30–39 year olds. In our series, the mean QuickDASH scores of patients for the different surgery types revealed no significant disability or slight disability compared with the general population. Similar results of favorable functional scores were found in the studies of Sahito et al. [15] and Papanastassiou et al. [36] In the Sahito et al. [15] study, no significant differences in long-term outcomes were noted between patients that received ECU tenodesis versus those without tenodesis (MWS, 91 vs 89; MSTS, 29 vs 29.2). This result was similar to that of the Papanastassiou et al. [36] study in which no obvious disparity in functional outcomes were revealed using QuickDASH or MSTS. One explanation for these results and ours is that these tests are not sufficiently sensitive, suffering to a certain extent from ceiling effects.

Surgeons might consider the Sauvé–Kapandji procedure to have theoretical advantages over the Darrach procedure, the latter of which has been reported to be associated with complications like painful proximal ulna stump instability, ulnar carpal shift, and complaints about esthetic appearance [37]. However, our results do not entirely support this notion. In our series, there was no radiological evidence of radioulnar convergence, and only one patient receiving the Darrach procedure experienced ulnar translocation of the carpus. Tomori et al. [13] reported that ECU tenodesis could not correct dorsal ulnar deviation or dorsal displacement of the radius and that proximal ulnar stump pain might be caused by dynamic factors rather than radial or dorsal deviation. Besides, in our series, re-operations were required as a result of surgical complications in 2 of 11 patients in the group receiving the modified Sauvé–Kapandji procedure with ECU tenodesis; these re-operations were necessary for reasons other than recurrence. Notably, the esthetic outcome scores in the six distinct subscales of MHQ for intralesional curettage and the Darrach procedure showed a significant difference, likely due primarily to the deformity of narrowing wrists after receiving the Darrach procedure. As the prominence of the “ulnar head” is maintained, satisfactory esthetic appearance was realized after the Sauvé–Kapandji procedure.

The present study has several limitations. These include its retrospective design and possible patient selection bias. Nevertheless, these issues are not uncommon for studies of patients with musculoskeletal tumors. Moreover, we did not include specific reconstruction types such as ulnar head arthroplasty. Thus, our ability to assess different reconstruction methods was limited.

## Conclusions

*En bloc* resection for distal ulna GCTB in our series showed a significantly lower recurrence rate compared with intralesional curettage and achieved favorable functional outcome scores. Given the higher recurrence rate after curettage, patients should be well informed of the potential benefits and risks of this DRUJ-preserving procedure. Moreover, our results suggest that reconstructions after tumor resection of the ulnar head do not appear to be necessary.

### Supplementary Information

**Additional file 1.** Patients’ detailed demographic information, and treatment modalities.

## Abbreviations

GCTB: Giant cell tumor of bone
ECU: Extensor carpi ulnaris
TFCC: Triangular fibrocartilage complex
MSTS: Musculoskeletal Tumor Society Scoring System
MWS: Mayo Wrist Score
MHQ: Michigan Hand Outcomes Questionnaire
DASH: Disabilities of the Arm, Shoulder, and Hand
ANOVA: Analysis of variance
ADL: Activities of daily living
DRUJ: Distal radioulnar joint
