# Supplementary material for: Giant cell tumors of the distal ulna: long-term recurrence rate and functional outcomes of en bloc resection versus curettage in a multicenter study
Source: J Orthop Surg Res. 2023 Sep 30;18:743. doi: 10.1186/s13018-023-04150-4 (PMC10544164; doi:10.1186/s13018-023-04150-4)
Supplement: Supplementary file 1 — Additional file 1. Patients’ detailed demographic information, and treatment modalities. [file 13018_2023_4150_MOESM1_ESM.docx]

**Table.** Patients’ detailed demographic information, and treatment modalities.

| **Case number/Gender/Age at diagnosis (years) /Side** | **Campanacci grading** | **Tumor size (cm)** | **Primary or recurrent** | **Fracture** | **Initial treatment** | **Further treatment** | **Time of recurrence (months)** | **Complications** | **Follow-up (months)** |
| --- | --- | --- | --- | --- | --- | --- | --- | --- | --- |
| 1/F/20/R | III | 3.7 | Recurrent | No | S-K+ECU |  |  |  | 25 |
| 2/F/21/L | III | 3.1 | Primary | No | S-K+ECU |  |  |  | 98 |
| 3/M/44/L | II | 2.5 | Primary | No | Curettage | Darrach | 37 |  | 188 |
| 4/F/30/L | II | 2.5 | Primary | Yes | Darrach |  |  |  | 124 |
| 5/M/36/R | II | 2.4 | Primary | No | Curettage | S-K+ECU | 131 |  | 142 |
| 6/M/57/L | III | 5.2 | Primary | Yes | S-K |  |  | Symptom of dorsal sensory branch of the ulnar nerve irritation | 24 |
| 7/F/32/L | III | 2.8 | Primary | No | S-K+ECU |  |  |  | 98 |
| 8/M/29/L | II | 2.3 | Primary | No | Curettage |  |  | Bone graft rejection and wound infection | 156 |
| 9/F/53/L | III | 2.4 | Primary | No | Darrach |  |  |  | 176 |
| 10/F/17/R | III | 3.5 | Primary | No | S-K+ECU | Screw removal |  | Screw looseness | 119 |
| 11/F/32/L | III | 3.3 | Primary | No | S-K+ECU | Screw removal |  | Resorption of autogenous iliac bone graft | 94 |
| 12/F/48/L | III | 3.1 | Recurrent | No | S-K+ECU |  |  |  | 50 |
| 13/M/38/L | III | 2.7 | Primary | Yes | S-K |  |  |  | 53 |
| 14/M/29/R | III | 3.6 | Primary | No | Curettage | S-K+ECU | 17 |  | 47 |
| 15/M/57/R | III | 4.7 | Recurrent | No | S-K+ECU |  |  |  | 94 |
| 16/F/40/R | II | 2.8 | Primary | No | Curettage |  |  |  | 80 |
| 17/F/24/R | III | 2.3 | Primary | No | Darrach |  |  |  | 169 |
| 18/M/25/L | III | 3.5 | Primary | No | S-K+ECU |  |  | Symptom of dorsal sensory branch of the ulnar nerve irritation | 76 |
| 19/M/28/L | III | 8.3 | Primary | No | S-K |  |  |  | 47 |
| 20/F/27/L | II | 2.6 | Primary | No | Curettage |  |  |  | 37 |
| 21/F/18/R | III | 2.5 | Primary | Yes | Darrach |  |  | Dorsal displacement of the distal ulnar stump | 100 |
| 22/F/23/L | II | 2.2 | Primary | No | S-K+ECU |  |  |  | 86 |
| 23/F/34/R | III | 2.6 | Primary | No | S-K |  |  |  | 27 |
| 24/M/25/R | III | 3.1 | Primary | Yes | Darrach | Resection for soft tissue recurrence | 31 |  | 85 |
| 25/F/29/L | II | 2.8 | Primary | No | Darrach |  |  |  | 87 |
| 26/M/28/L | II | 2.4 | Primary | No | Darrach |  |  |  | 63 |
| 27/M/47/R | III | 2.1 | Primary | Yes | Curettage |  |  |  | 64 |
| 28/F/24/R | II | 2.4 | Primary | Yes | S-K |  |  |  | 77 |
